# Glutathione depletion induces ferroptosis, autophagy, and premature cell senescence in retinal pigment epithelial cells

**DOI:** 10.1038/s41419-018-0794-4

**Published:** 2018-07-09

**Authors:** Yun Sun, Yingfeng Zheng, Chunxiao Wang, Yizhi Liu

**Affiliations:** State Key Laboratory of Ophthalmology, Zhongshan Ophthalmic Center, Sun Yat-sen University, Guangzhou, China

## Abstract

Glutathione (GSH) protects against oxidative damage in many tissues, including retinal pigment epithelium (RPE). Oxidative stress-mediated senescence and death of RPE and subsequent death of photoreceptors have been observed in age-related macular degeneration (AMD). Although the consequences of GSH depletion have been described previously, questions remain regarding the molecular mechanisms. We herein examined the downstream effects of GSH depletion on stress-induced premature senescence (SIPS) and cell death in human RPE cells. Briefly, cultured ARPE-19 cells were depleted of GSH using: (1) incubation in cystine (Cys_2_)-free culture medium; (2) treatment with buthionine sulphoximine (BSO, 1000 µM) to block de novo GSH synthesis for 24–48 h; or (3) treatment with erastin (10 µM for 12–24 h) to inhibit Cys_2_/glutamate antiporter (system x_c_^−^). These treatments decreased cell viability and increased both soluble and lipid reactive oxygen species (ROS) generation but did not affect mitochondrial ROS or mitochondrial mass. Western blot analysis revealed decreased expression of ferroptotic modulator glutathione peroxidase 4 (GPX4). Increased autophagy was apparent, as reflected by increased LC3 expression, autophagic vacuoles, and autophagic flux. In addition, GSH depletion induced SIPS, as evidenced by increased percentage of the senescence-associated β-galactosidase-positive cells, increased senescence-associated heterochromatin foci (SAHF), as well as cell cycle arrest at the G1 phase. GSH depletion-dependent cell death was prevented by selective ferroptosis inhibitors (8 μM Fer-1 and 600 nM Lip-1), iron chelator DFO (80 μM), as well as autophagic inhibitors Baf-A1 (75 nM) and 3-MA (10 mM). Inhibiting autophagy with Baf-A1 (75 nM) or 3-MA (10 mM) promoted SIPS. In contrast, inducing autophagy with rapamycin (100 nM) attenuated SIPS. Our findings suggest that GSH depletion induces ferroptosis, autophagy, and SIPS. In addition, we found that autophagy is activated in the process of ferroptosis and reduces SIPS, suggesting an essential role of autophagy in ferroptosis and SIPS.

## Introduction

The eyes are exposed to constant irradiation, and therefore have extraordinary need for antioxidant protection^1^. RPE is particularly susceptible to oxidative stress due to its role in phagocytosis of photoreceptor outer segments^2^. Phagocytosed photoreceptor outer segments are highly enriched in polyunsaturated fatty acids and are the major source of intracellular ROS generation in RPE^3,4^. In addition, partial oxygen pressure in RPE is high due to anatomical proximity to choriocapillaries^5–7^. Studies using exogenous oxidants such as *tert*-butyl hydroperoxide (tBH) and hydrogen peroxide (H_2_O_2_) in cultured RPE cells highlight the role of oxidative stress in RPE cell death and premature senescence^8–11^. However, it is unclear to what extent the in vitro observations correspond to metabolic and disease processes seen in vivo^12^.

GSH is the most prominent antioxidant in RPE cells and is present at high concentration in retina and RPE^13,14^. However, the efficiency of GSH redox system declines with age, predisposing the RPE to increased oxidative-stress-mediated damage^15,16^. Exogenously administered GSH protects against oxidative damage in cultured human RPE^17^, while GSH depletion was shown to cause cell death^18^. However, the mechanism beneath the effect of GSH depletion-induced oxidative stress in RPE cells and the subsequent RPE damage is not well established. Therefore it is essential to investigate the effect of GSH depletion on RPE cells to further elucidate the potentially involved mechanisms.

Cell death types induced by ROS include apoptotic, autophagic, ferroptotic, and necrotic cell deaths. Earlier studies demonstrated that GSH depletion could induce apoptosis^19,20^ or necrosis^21^ in RPE cells. In other cell types such as spermatogonial cells, GSH depletion may induce autophagy^22^. Recent reports demonstrated that GSH depletion triggers ferroptosis in some cell types^18^. It is likely that variations in experimental methods, biological systems, as well as the distinct metabolic characteristics of cells contributed to the variability in reported results^18,23^. Ferroptosis, a form of regulated cell death initiated by lipid peroxidation, differs from other types of cell deaths at genetic, biochemical, and morphological levels^24,25^. Ferroptosis is regulated by distinct molecular pathways and was shown to play an important role in degenerative and neoplastic diseases^26^. Nonetheless, whether ferroptotic cell death is associated with the effects of GSH depletion on RPE is unknown.

Besides cell death, ROS generated from oxidative stress also triggers SIPS. SIPS is a phenomenon characterized by the irreversible cessation of the division of normal cells even in presence of nutritional and mitogenic factors^27^. SIPS can be caused by various factors including persistent exposure to cell stress, namely, oxidative stress or DNA-damaging mediators^28,29^. Documented data indicate that premature senescence of ARPE-19 cells is possibly involved in the features of AMD^10,28^. Previous studies reported that SIPS can be triggered by oxidative stress caused by tBH and H_2_O_2_^10,11^; however, whether GSH depletion is linked with SIPS in RPE is unknown.

Hence, in this study, we aimed to investigated the effect of GSH depletion-induced oxidative stress in RPE cells. We also sought to explore the nature of cell death involved and examine whether RPE cells experience SIPS following GSH depletion.

## Results

### GSH depletion induces cell death in RPE cells

**Fig. 1:**
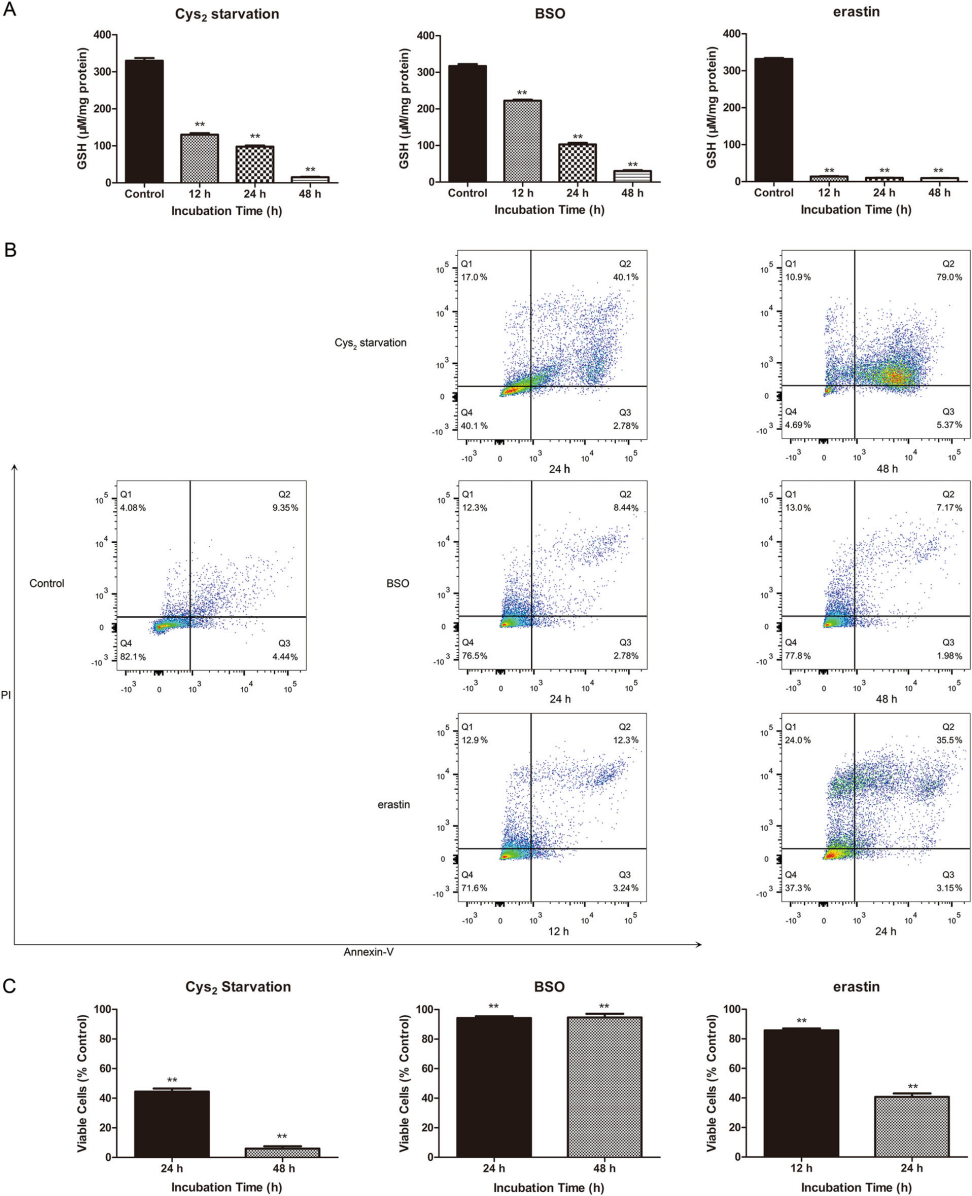
GSH depletion induces cell death in RPE cells. **a** Quantification of intracellular GSH content. Cells were treated with Cys_2_ starvation, 1000 µM BSO or 10 µM erastin for 12, 24, and 48 h, respectively. **b** GSH depletion-induced cell death in ARPE-19 cells revealed by flow cytometry. The upper panel: Cys_2_ starvation for 24 and 48 h. The middle panel: BSO (1000 µM) treatment for 24 and 48 h. The lower panel: erastin (10 µM) treatment for 12 and 24 h. Quadrant Q4: viable cells. Numbers displayed in each quadrant represent proportion of cells. **c** Represent quantification of the effects depicted in (**b**). Data in (**a**) and (**c**) represent mean ± SD from one of three representative experiments. ** represent *p* < 0.01

To evaluate the effect of GSH depletion on the RPE cells, we used three different approaches: depletion of Cys_2_ from cell culture media, treatment with buthionine sulphoximine (BSO, 1000 µM) to block de novo synthesis of GSH, or treatment with erastin (10 µM), an inhibitor of system x_c_^−^^1^. GSH depletion was apparent after all three treatments (Fig. 1a), and all treatments lead to decreased cell viability, as measured by annexin V-PI assay (Fig. 1b, c).

### GSH depletion causes oxidative stress and lipid peroxidation

**Fig. 2:**
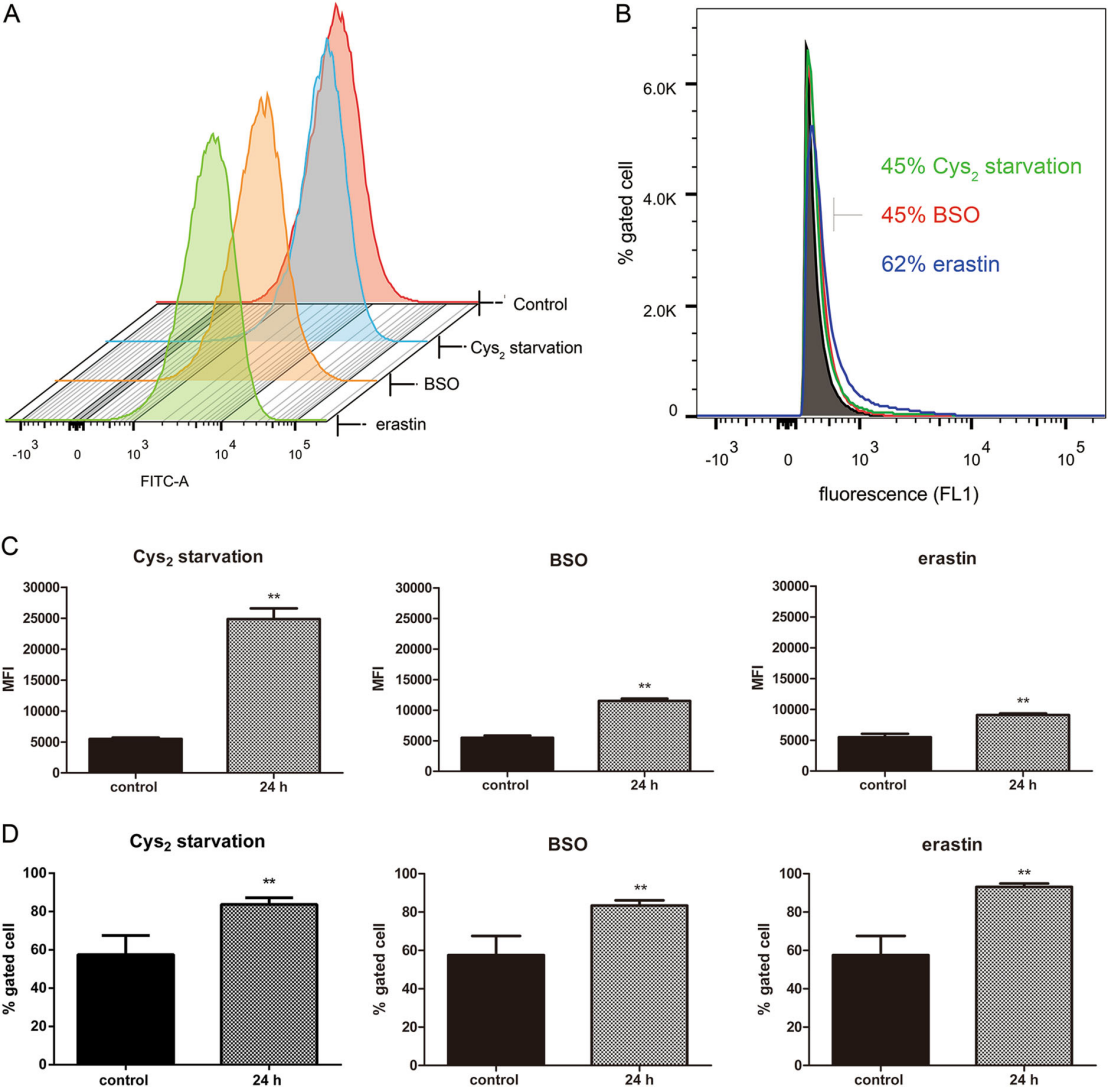
GSH depletion induces oxidative stress and lipid peroxidation in RPE cells. **a** Cytosolic ROS production assessed in ARPE-19 cells treated for 24 h with Cys_2_ starvation, 1000 µM BSO, and 10 µM erastin by flow cytometry using H_2_DCFDA. **b** Lipid ROS production assessed in ARPE-19 cells treated for 24 h with Cys_2_ starvation, 1000 µM BSO, and 10 µM erastin by flow cytometry using C11-BODIPY. **c**, **d** represent quantified data depicted in (**a**, **b**). Data in (**c**, **d**) represent mean ± SD from one of three representative experiments. Representative data from one of three experiments are shown. ** represent *p* < 0.01

To evaluate the downstream effects of GSH depletion, we measured soluble ROS in RPE cells treated with Cys_2_ starvation, BSO, or erastin by flow cytometry using the fluorescent probe H_2_DCFDA. GSH depletion increased cytosolic ROS (Fig. 2a, c). To determine the site of ROS generation, redox-sensitive dye BODIPY 581/591 C11 was used to detect lipid ROS (Fig. 2b). Stimulation of BODIPY-loaded RPE cells with Cys_2_ starvation or with BSO/erastin treatment increased ROS generation as measured by increased rate of BODIPY oxidation (Fig. 2d).

### GSH depletion does not induce mitochondrial superoxide or alter mitochondrial mass in RPE cells

**Fig. 3:**
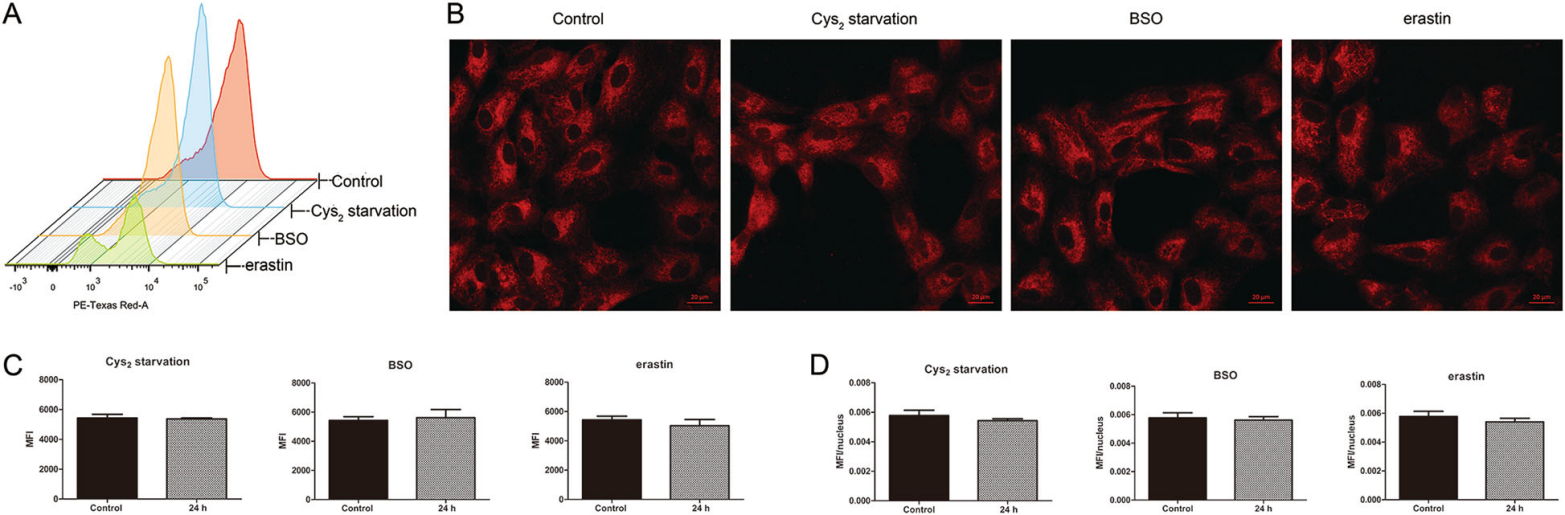
GSH depletion does not induce mitochondrial superoxide or alter mitochondrial mass in RPE cells. **a** Mitochondrial ROS assessed in ARPE-19 cells treated for 24 h with Cys_2_ starvation, 1000 µM BSO, and 10 µM erastin by flow cytometry using MitoSOX Red. **b** Mitochondrial mass was measured by MitoTracker Red dye. Scale bars: 20 μm. (**c**) and (**d**) represent quantified data depicted in (**a**, **b**). Data in (**c**, **d**) represent mean ± SD from one of three representative experiments. Representative data from one of three experiments are shown

It has been suggested that mitochondria are predominant source of ROS upon H_2_O_2_-induced oxidative stress^7^. We next investigated whether mitochondria are the source of ROS generation in RPE cells upon GSH depletion, we used MitoSOX Red to detect mitochondrial ROS (Fig. 3a, c). Stimulation of MitoSOX-loaded RPE cells with Cys_2_ starvation or BSO did not cause oxidation of the fluorophore. We also failed to detect an increase in MitoSOX-sensitive mitochondrial ROS production in erastin-treated RPE cells. These observations are in line with the previous findings of Dixon et al. in HT-1080 cancer cells where treatment with erastin did not cause mitochondrial ROS production^24^. It has been demonstrated that oxidative stress and excessive generation of ROS affect mitochondrial morphology^30^. To determine if GSH depletion-induced oxidative stress impairs mitochondrial biogenesis, we used MitoTracker Red to stain live mitochondria. We calculated the mean fluorescent intensity (MFI) using ImageJ software as described in Methods. Cells subjected to Cys_2_ starvation or BSO and erastin coincubation did not alter MitoTracker fluorescence intensity (Fig. 3b, d). These findings further ruled out mitochondrial damage to be the source of GSH depletion-induced oxidative stress.

### GSH depletion causes ferroptosis in RPE cells

**Fig. 4:**
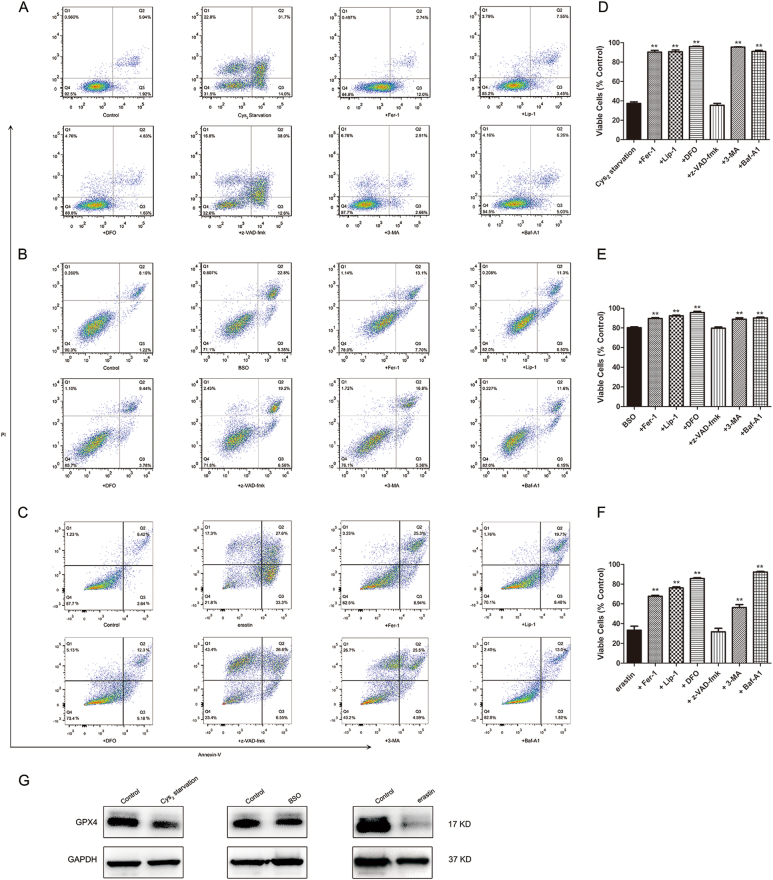
GSH depletion induces ferroptosis in RPE cells. **a**–**c** Cell viability detection with Annexin V/PI by flow cytometry. **a** Cys_2_ starvation with or without selected cell death inhibitors. **b** BSO (1000 µM) treatment with or without selected cell death inhibitors. **c** Erastin (10 µM) treatment with or without selected cell death inhibitors. Ferroptosis-specific inhibitor Fer-1 (8 μM), Lip-1 (600 nM), iron chelator DFO (80 μM), pan-caspase inhibitor z-VAD-fmk (30 μM), autophagic inhibitor 3-MA (10 mM), and lysosomal inhibitor Baf-A1 (75 nM) were diluted in Cys_2_-free culture medium or coincubated with BSO or erastin treatment at indicated dose (all stock dissolved in DMSO except 3-MA; the latter is water soluble) for 24 h. Quadrant Q4: viable cells. Numbers displayed in each quadrant represent proportion of cells. (**d**–**f**) represent quantified data depicted in (**a**–**c**). Data in (**d**–**f**) represent mean ± SD from one of three representative experiments. Representative data from one of three experiments are shown. ** represent *p* < 0.01. **g** GPX4 downregulation as assessed by immunoblotting

To evaluate the mechanism of cell death in GSH-depleted cells, we treated RPE cells with various cell death pathway inhibitors including ferroptosis inhibitors ferrostatin-1 (Fer-1, 8 μM), liproxstatin-1 (Lip-1, 600 nM), iron chelator deferoxamine (DFO, 80 μM), pan-caspase inhibitor z-VAD-fmk (30 μM) to inhibit apoptosis, autophagy inhibitor 3-methyladenine (3-MA, 10 mM), and lysosomal inhibitor bafilomycin A1 (Baf-A1, 75 nM). These reagents were added either in Cys_2_-free culture medium or coincubated with BSO or erastin at indicated concentrations. Annexin V-PI apoptosis assay was performed following the treatments to assess cell viability (Fig. 4a–c).

Cell death inhibitors achieved comparable effects in Cys_2_-starved or BSO/erastin-treated RPE cells. Fer-1 increased viability to 90% of cells under Cys_2_ starvation and in BSO-treated cells, and to 70% in erastin-treated cells (Fig. 4d–f, *p* < 0.01). Lip-1 increased viability to 90% under Cys_2_ starvation and in BSO-treated cells, and to 70% in erastin-treated RPE cells (Fig. 4d–f, *p* < 0.01). Similar protective effects were observed with DFO (Fig. 4d–f, *p* < 0.01). No rescuing effects were observed with z-VAD-fmk, suggesting that apoptosis is not induced by GSH depletion. Intriguingly, lysosomal and autophagy flux inhibitor Baf-A1 showed similar rescuing effect as DFO. Another autophagy inhibitor 3-MA also produced rescuing effects but to a smaller extent in erastin-treated cells. These findings suggest that cell death in GSH-depleted cells happens through ferroptosis and autophagy. Ferroptosis is regulated by GPX4, an enzyme that reduces lipid hydroperoxides within biological membranes^26^. Inactivation of GPX4 leads to accumulation of lipid ROS and induces ferroptosis^18,31^. We found that expression of GPX4 was reduced after Cys_2_ starvation and erastin treatment, further proving that ferroptosis is a primary mechanism of GSH depletion-induced cell death in RPE (Fig. 4g).

### GSH depletion triggers autophagy

**Fig. 5:**
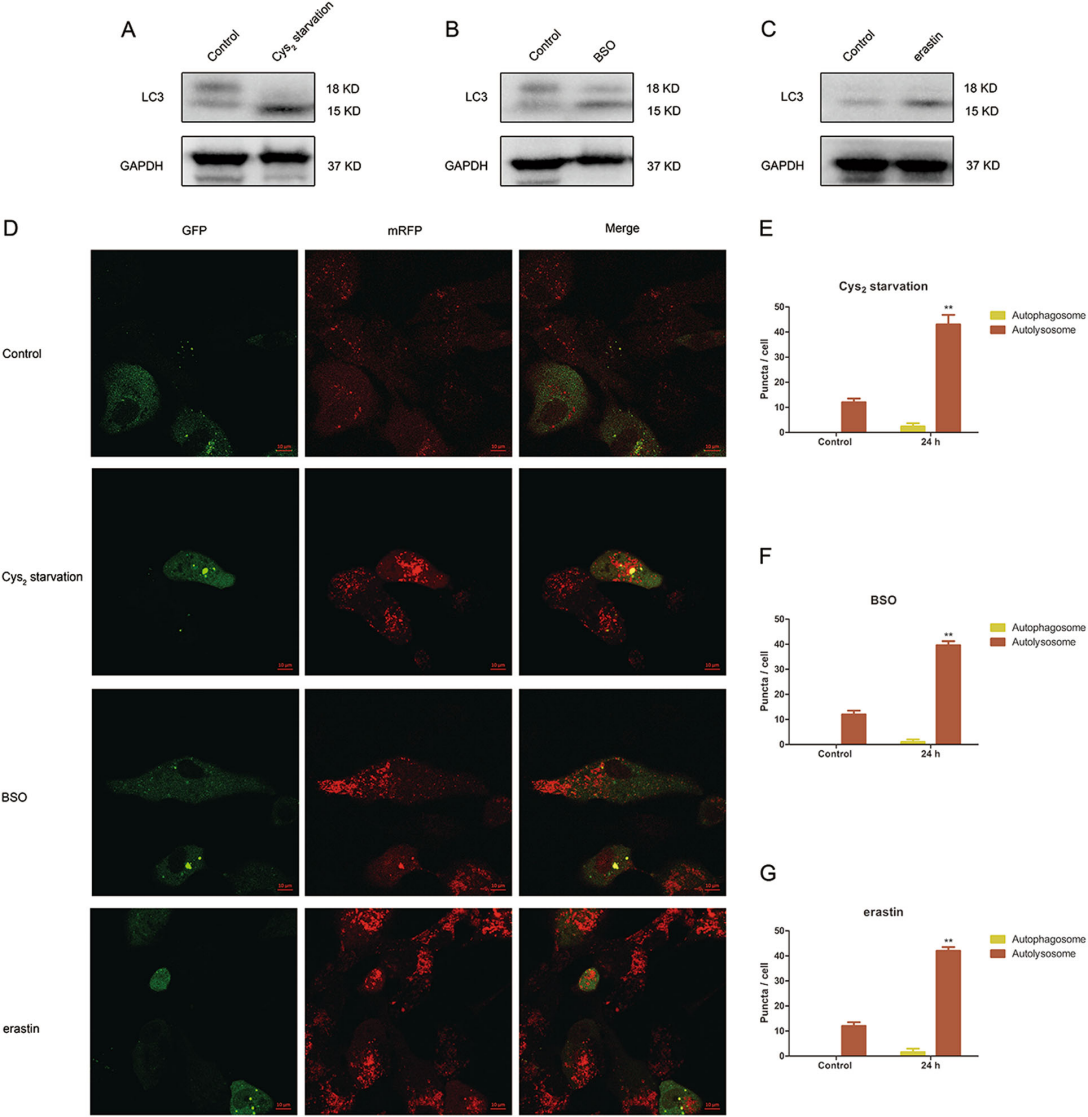
GSH depletion triggers autophagic activation accompanied by an increasing autophagic flux. (**a**–**c**) represent autophagy activation in Cys_2_ starved, 1000 µM BSO, and 10 µM erastin-treated RPE cells, respectively, by detect LC3 using western blot. **d** After being exposed to Cys_2_ starvation milieu, 1000 µM BSO and 10 µM erastin for 24 h, representative images of ARPE-19 cells displaying LC3 puncta were immediately visualized by confocal microscopy. Number of autophagosomes represented by yellow puncta and autolysosomes represented by red puncta in merged images. Scale bar, 10 µm. (**e**–**g**) represent quantified data depicted in (**d**). Red puncta and yellow puncta were counted and calculated as number per cell. Data represent mean ± SD from one of three representative experiments. Representative data from one of three experiments are shown. ** represent *p* < 0.01

As autophagy inhibitors Baf-A1 and 3-MA protected from cell death in response to GSH depletion, we investigated whether autophagy is induced by GSH depletion. Western blot analysis of LC3II, a surrogate marker for autophagy^32^, revealed autophagic induction in GSH-depleted cells. In comparison to the controls, LC3-II level was increased in Cys_2_-starved, BSO, and erastin-treated cells (Fig. 5a–c).

Next, we monitored autophagic flux in GSH-depleted cells by using a tandem fluorescent-tagged LC3 lentivirus mRFP-GFP-LC3 expression system. mRFP-GFP-LC3 is a ratiometric probe bound to both the inner and outer membrane of the autophagosome^33^. GFP signals within the inner autophagosome membrane are degraded in the autolysosomes but mRFP signals are relatively stable and thus provide a visible measure of autophagic flux status ^33^.

mRFP-GFP-LC3 lentivirus were transduced into RPE cells prior to treatment. Red puncta (mRFP only, representing autolysosomes) and yellow puncta (overlapping of mRFP and GFP, representing autophagosomes) were counted and presented as number per cell. GSH depletion in RPE cells increased the number of red puncta, indicating an increase in autophagic flux (Fig. 5d–g). The observed effect was least pronounced in the BSO-treated group, as shown in western blot.

**Fig. 6:**
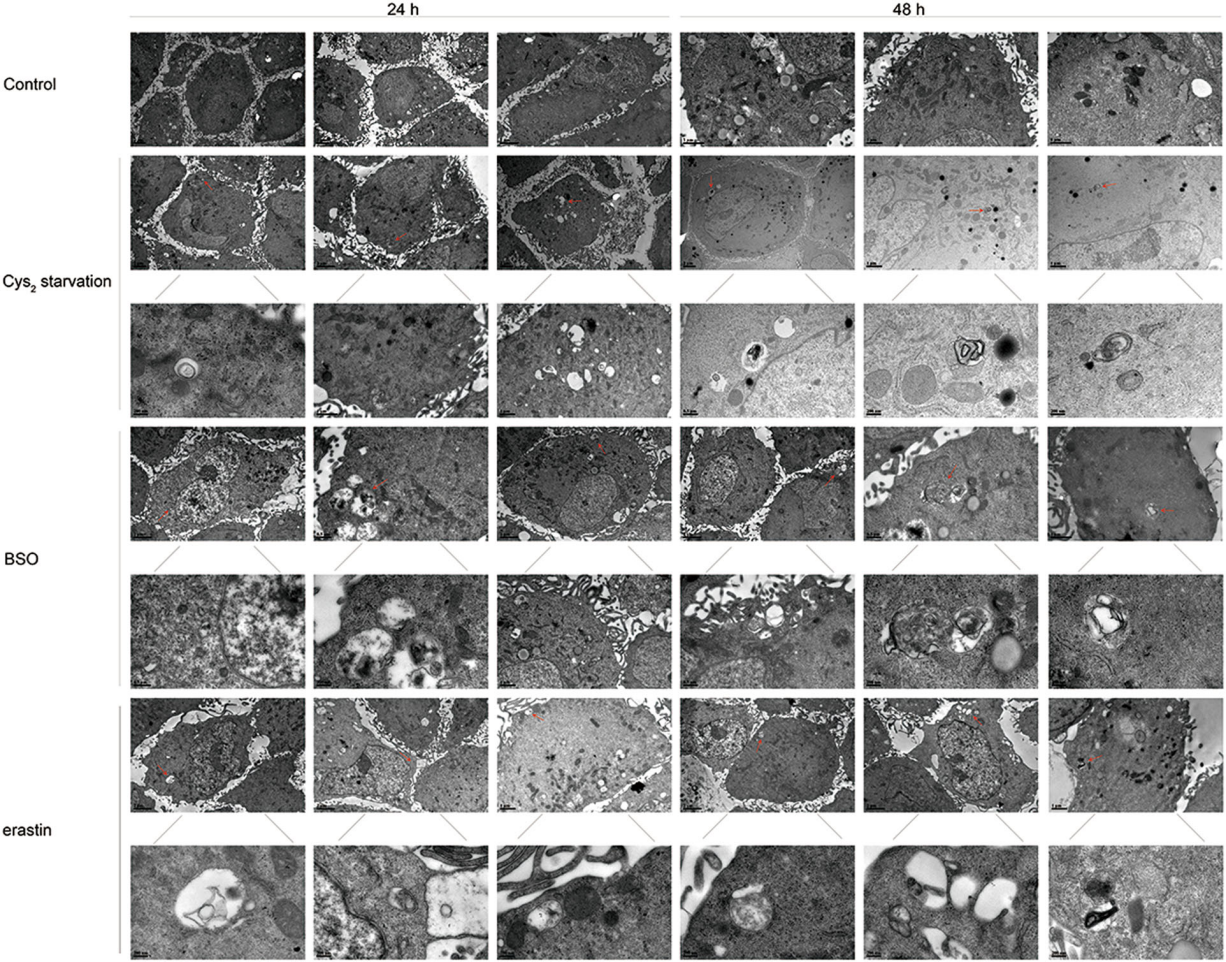
Ultrastructure of GSH depleted RPE cells. Representative TEM photomicrographs of ARPE-19 cells exposed to Cys_2_ starvation milieu, 1000 µM BSO, and 10 µM erastin for 24 and 48 h. Autophagic vacuoles are indicated by red arrows. The lower panel of each treatment shows the high magnification image of the upper panel. Scale bars are displayed in each image

Finally, we performed transmission electron microscopy (TEM) to detect autophagic vesicles at the ultrastructural level. Autophagosomes appearing as double membrane structures were observed in cells treated with Cys_2_ starvation, BSO, and erastin (Fig. 6).

### GSH depletion triggers cell cycle arrest in G1/S checkpoint and induces SIPS

**Fig. 7:**
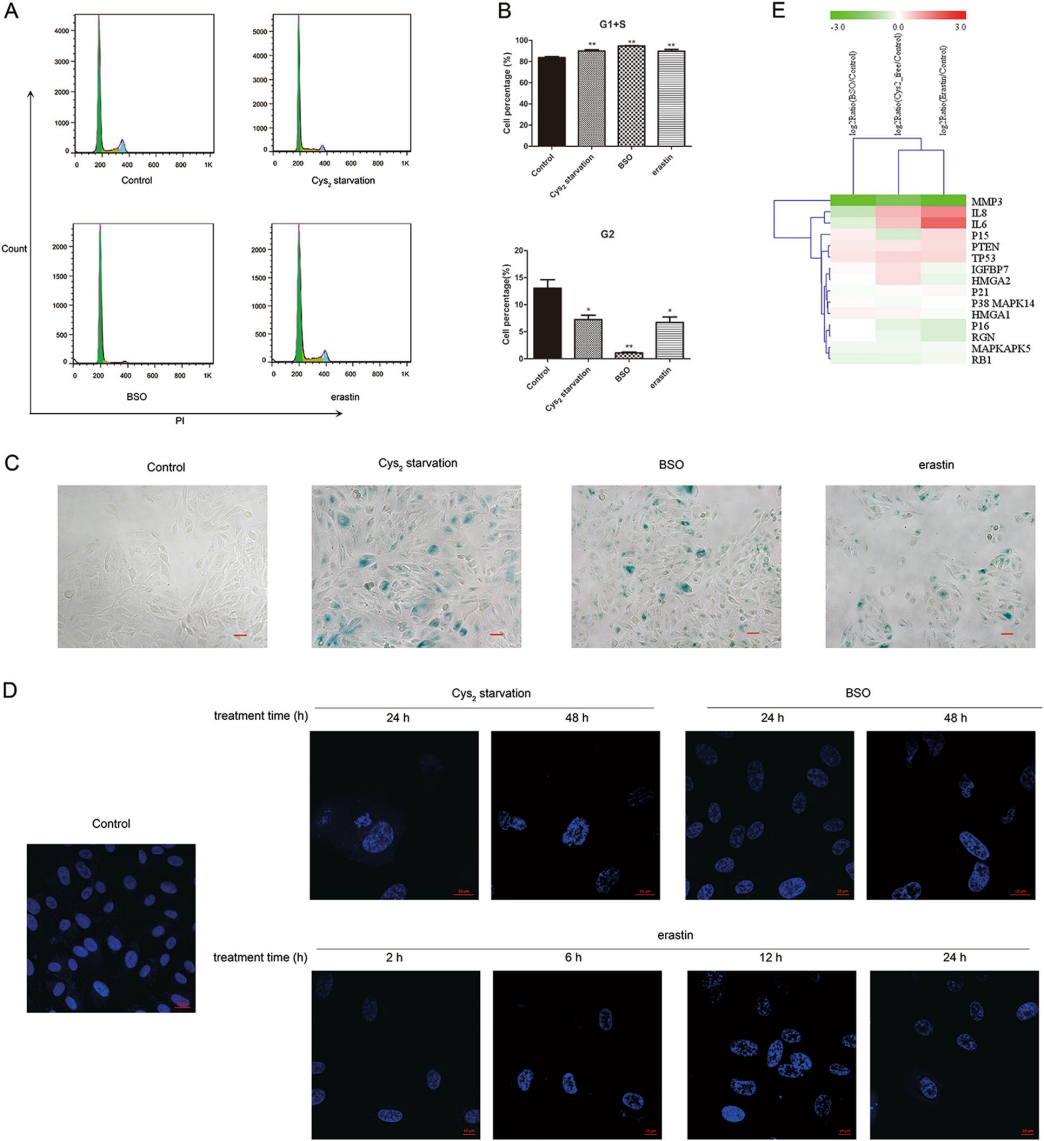
GSH depletion arrests cell growth and induces premature cell senescence. **a** Cell cycle analysis by PI staining in cells treated with Cys_2_ starvation, 1000 µM BSO, and 10 µM erastin. (**b**) represent quantified data depicted in (**a**). Proportion of cells in G1 + S phase and G2 phase were calculated. Data represent mean ± SD from one of three representative experiments. Representative data from one of three experiments are shown. * represent *p* < 0.05, ** represent *p* < 0.01. **c** Images of senescence β-galactosidase staining. Cells were treated with Cys_2_ starvation, 1000 µM BSO, and 10 µM erastin for 24 h, respectively. Scale bars: 50 μm. **d** GSH-depleted cells were stained with DAPI to show SAHFs. **e** Senescence-associated gene profiles of Cys_2_ starved and 1000 µM BSO and 10 µM erastin-treated ARPE-19 cells. Total RNA was extracted at 24 h post treatment and assayed on BGISEQ-500 RNA-Seq

SIPS is well-characterized in cells treated with exogenous oxidants such as tBH and H_2_O_2_^10,11^. Features of SIPS include increased senescence-associated β-galactosidase (SA-β-Gal) activity, appearance of senescence-associated heterochromatin foci (SAHF), cell growth arrest, and increased expression of senescence-associated genes^11,34^. To examine whether GSH depletion causes SIPS in RPE cells, we first analyzed cell cycle by flow cytometry. As shown in Fig. 7a, b, Cys_2_ starvation as well as BSO and erastin treatment increased the percentage of cells in the G0/G1 phase with a concomitant decrease in the G2 phases, suggesting that GSH depletion induces growth arrest in RPE cells.

Next, SA-β-Gal staining was performed to monitor SIPS induction. GSH depletion increased the percentage of SA-β-Gal-positive cells (Fig. 7c). SAHFs in these cells were evident as large and irregularly shaped nuclear puncta (Fig. 7d). In addition, senescence-associated secreted factors, such as interleukin 6 (IL-6), interleukin 8 (IL-8), also displayed similar expression profiles, particularly in Cys_2_-starved and erastin-treated cells (Fig. 7e). These observations suggested that GSH depletion induces SIPS in RPE cells.

### Rapamycin-induced autophagy ameliorates GSH depletion-induced premature cell senescence

It was shown that autophagy activation especially in the presence of an increasing autophagic flux inhibits the senescence of RPE cells^35–39^. In our study, we observed the senescence of RPE cells despite the concomitant induction of autophagy following GSH depletion. To determine if pharmacological manipulation of autophagy effects SIPS, we treated GSH-depleted cells with the autophagic inducer rapamycin and autophagic inhibitors 3-MA and Baf-A1.

**Fig. 8:**
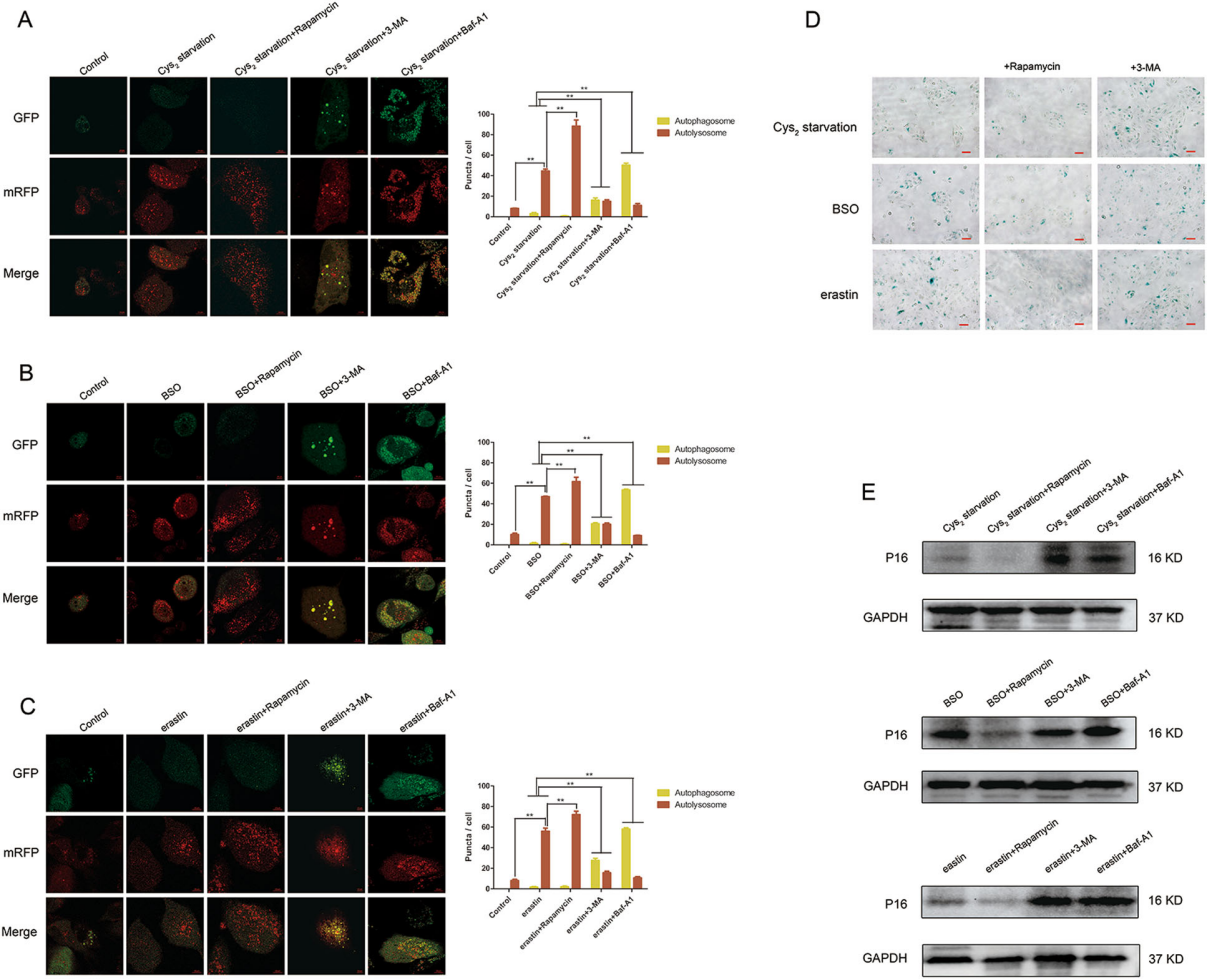
Rapamycin-induced autophagy ameliorates GSH depletion-induced premature cell senescence. **a**–**c** ARPE-19 cells were coincubated with Cys_2_ starvation milieu, 1000 µM BSO, and 10 µM erastin supplied with autophagic inducer rapamycin (100 nM), autophagic inhibitor 3-MA (10 mM) and lysosomal inhibitor Baf-A1 (75 nM) for 24 h; representative images of RPE cells displaying LC3 puncta were immediately visualized by confocal microscopy. Mean number of autophagosomes represented by yellow puncta in merged images and autolysosomes represented by red puncta in merged images per cell. Scale bar, 10 µm. ** represent *p* < 0.01. **d** Images of senescence β-galactosidase staining. Cells were treated with Cys_2_ starvation, 1000 µM BSO, and 10 µM erastin with or without 100 nM rapamycin and 10 mM 3-MA for 24 h, respectively. Scale bars: 50 μm. **e** Downregulation of p16 after coincubation with autophagic inducer rapamycin, while upregulation of p16 after coincubation with autophagic inhibitor 3-MA and lysosomal inhibitor Baf-A1 were detected by western blotting

Autophagic flux tracing using tandem mRFP-GFP-LC3 probe showed that increase of autophagic flux caused by GSH depletion is augmented by rapamycin and attenuated by autophagic inhibitors 3-MA and Baf-A1 (Fig. 8a–c).

SA-β-Gal staining showed that rapamycin decreased senescence, whereas 3-MA promoted senescence (Fig. 8d). Mechanistically, oxidative stress activates p16-pRb effector pathway which is thought to play a vital role in SIPS mediation^11^. Upregulation of p16 leads to the hypophosphorylation of the pRb protein and induction of senescence^11,40^. In cells depleted of GSH, p16 expression was significantly decreased by rapamycin and increased by 3-MA and Baf-A1 (Fig. 8e).

## Discussion

Our results suggest that GSH depletion induces ferroptosis, autophagy, and SIPS in RPE cells. First, we characterized the mechanism of cell death observed in GSH-depleted RPE through a series of functional measurements. Second, the level of autophagy was analyzed in GSH-depleted RPE cells. Third, we found that GSH depletion induced SIPS. The interaction between autophagy and SIPS was analyzed using pharmacological autophagy modulators.

Ferroptosis is characterized by iron-dependent accumulation of lipid ROS and is distinct morphologically and mechanistically from apoptosis or other programmed cell death pathways^41,42^. Although the physiological function of ferroptosis remains poorly defined, it has been shown to be involved in various diseases^43–45^. This study is the first to describe the relationship between ferroptosis and GSH depletion in RPE cells. GSH serves as a direct antioxidant as well as an important substrate for antioxidant GPX4 to prevent lipid ROS accumulation; thus, GSH depletion ultimately leads to increased lipid peroxidation^23^. GSH depletion has been classified as type 1 ferroptosis inducer^44,46^. However, it was shown that GSH depletion induces ferroptosis in some cell types but not others^23^. The discrepancy may be attributed to the distinct metabolic characteristics of cells and the variations in the induction approaches used in different studies^18,23^. It was shown that in some cells direct inhibition of glutamate cysteine ligase (GCL) by BSO may upregulate factors involved in other antioxidative pathways (e.g. GSH-independent thioredoxin pathway), which may prevent GSH depletion-dependent oxidative stress and cell death^18^. In addition, some cell types can utilize transsulfuration and use methionine to biosynthesize cysteine. Thus, in these cells, Cys_2_ starvation or system x_c_^−^ inhibitor (such as erastin) cannot induce oxidative stress and cell death^26^. Therefore, GSH depletion-induced oxidative stress and cell death depend on the combination of inducer and cell-specific molecular characteristics, i.e. inability to synthesize GSH and absence of alternative GSH-independent antioxidative systems.

In the current study, depriving RPE cells of the essential GSH precursor Cys_2_ by culturing cells in Cys_2_-free culture medium or blocking de novo synthesis of GSH by using GCL inhibitor BSO or blocking Cys_2_ uptake by pharmaceutical inhibition of system x_c_^−^ caused cell death. However, BSO induced cell death to a lesser extent with slower kinetics. We demonstrated that the ferroptosis inhibitors Fer-1 and Lip-1 protected against cell death induced by GSH depletion. Fer-1 is a potent ferroptosis inhibitor that prevents accumulation of cytosolic and lipid ROS^24^. The ferroptosis inhibitor Lip-1 functions as a lipophilic antioxidant similar to Fer-1^44^. In addition, we showed that iron chelator DFO also rescued cells from Cys_2_ starvation and BSO/erastin-induced death. While the largest percentage of intracellular iron is tightly bound to or incorporated into proteins as a cofactor or for storage, small portion of intracellular iron resides in the cytosol and intracellular organelles (e.g., lysosomes) and constitutes a redox-active liable iron pool, which regulates programmed cell death including ferroptosis^42^. Since DFO is membrane impermeable and accumulates in lysosomes^18^, we believe that it protects the cells against ferroptosis by chelating lysosomal iron as described previously ^47^.

Lysosomal inhibitor Baf-A1 and autophagy inhibitor 3-MA also protected against cell death induced by GSH depletion, highlighting the role of autophagy in ferroptosis. Considering the fact that Baf-A1 suppresses fusion of autophagosomes with the lysosomes^39^, our finding that DFO protected against ferroptosis suggests that lysosomal iron pool is involved in GSH depletion-induced cell death^48^. Ferroptosis is a novel programmed cell death type distinct from autophagy^24^. However, emerging evidence indicated that ferroptosis is an autophagic cell death process^48–50^. Autophagy is a highly dynamic, multistep process involving the degradation of cytoplasmic cargo through the lysosomal machinery^51^ and serves as a protective response mechanism under oxidative stress^52^. Canonical autophagy machinery plays a crucial role in ferroptosis^48^. Autophagy and functional lysosomes probably contribute to ferroptosis through the provision of iron ^50^.

Mitochondria are the main source of ROS in response to oxidants^7^, and play dominant roles in apoptosis^53–58^ and necrosis in RPE cells^59,60^. Mitochondria are also involved in the induction of autophagy^61,62^. However, in our study, oxidative stress-induced cell death was triggered by lipid peroxidation instead of the canonical mitochondrial ROS accumulation. RPE is particularly prone to lipid peroxidation owing to its persistent phagocytosis of photoreceptor outer segments. Two major lipid peroxidation products, malondialdehyde (MDA) and 4-hydroxynonenal (4-HNE), increase with aging and contribute to the pathogenesis of AMD ^63–65^.

GSH appears to be involved in the crosstalk between ferroptosis and autophagy. Direct inhibition of GSH synthesis triggers ferroptosis^18^ and autophagy^22,66^. On the other hand, autophagy leads to a significant decrease in intracellular GSH levels and vice versa^67^. The underlying mechanism of how GSH modulate the complex crosstalk between ferroptosis and autophagy remains unclear. In view of the pivotal role played by GSH in redox system and the relationship between oxidative stress and autophagy^68^, it is conceivable that GSH depletion-induced oxidative stress and lipid peroxidation may be a key point linking ferroptosis and autophagy in RPE cells.

GSH depletion seemed to produce contradicting effects in our study. GSH depletion induced autophagy accompanied by ferroptosis, as well as cell senescence. The relationship among GSH depletion, ferroptosis, and senescence has not been reported. In our experiments, exogenous rapamycin significantly decreased the proportion of SA-β-Gal-positive cells and p16 expression, whereas autophagy inhibitors 3-MA and Baf-A1 produced opposite effects. The crosstalk between autophagy and senescence remains poorly defined. A number of studies have provided indirect or circumstantial evidence for the collateral induction of autophagy and senescence^69–72^. However, other reports supported an inverse relationship^35–38^. Senescent cells with impaired autophagy are highly resistant to ferroptosis^73^. In a recently published report, autophagic flux restoration ameliorated cell senescence^39^. The authors claimed that autophagic flux is decreased, rather than increased, by oxidative stress triggered by exogenous oxidants H_2_O_2_. Variation in the observed results could be attributed to the main source of ROS: mitochondria in their study vs. lipid peroxidation in our study.

In conclusion, the current study demonstrated, for the first time, that GSH depletion induces ferroptosis in RPE cells. In addition, we showed that GSH depletion induces autophagy and SIPS, with autophagy being a negative regulator of SIPS. However, due to the lack of long-term observation of GSH depletion and in vivo animal experiments, it is difficult to evaluate whether ferroptosis is a bona fide autophagy activation process or a prerequisite for autophagy activation process. Further studies are required to answer these questions and dissect the exact relationship between ferroptosis, autophagy, and SIPS in RPE. Despite the limitations, this study sheds a new light on GSH depletion-induced cell death and senescence.

## Material and methods

### Reagents and chemicals

Erastin, rapamycin, z-VAD-fmk, 3-MA, and DFO were purchased from Sigma-Aldrich (St. Louis, MO, USA). Fer-1, Lip-1, and Baf-A1 were purchased from Selleck Chemicals (Houston, TX, USA). BSO was purchased from Cayman Chemical (Ann Arbor, MI, USA). Anti-LC3B (D11), anti-GAPDH antibodies, and senescence β-galactosidase staining kit were purchased from Cell Signaling Technology (Danvers, MA, USA). Micro BCA Protein Assay Kit, H_2_DCFDA (H_2_-DCF, DCF), C11-BODIPY (581/591), MitoSOX™ Red Mitochondrial Superoxide Indicator, MitoTracker^®^ Red CMXRos, and Dulbecco’s modified Eagle’s medium (High glucose, no glutamine, no methionine, no Cys_2_) were purchased from Thermo Scientific (Waltham, MA, USA). Dulbecco’s modified Eagle’s medium/F12 and fetal bovine serum were purchased from Gibco (Logan, UT, USA). Anti-p16-INK4A antibodies was purchased from Proteintech (Rosemont, IL, USA). Alexa Fluor^®^ 488 annexin V/dead cell apoptosis kit was purchased from Invitrogen (Carlsbad, CA, USA). Anti-GPX4 antibody and propidium Iodide flow cytometry kit were purchased from Abcam (Cambridge, MA, USA). GSH assay kit was purchased from Beyotime Biotechnology (Nantong, Jiangsu, China).

### Cell culture and STR analysis

ARPE-19 human RPE cells were purchased from the American Type Culture Collection (ATCC, Manassas, VA, USA), and cultured in DMEM/F12 medium supplemented with 10% fetal bovine serum at 37 °C in air containing 5% CO_2_. The batch of the ARPE-19 cells used in this study was validated using short tandem repeat (STR) analysis by Cobioer Biosciences (Nanjing, China). Briefly, genomic DNA was extracted from the cell pellets and amplified using GenePrint System (Promega). Amplified products were processed using the ABI3730xl Genetic Analyzer. Data were analyzed using GeneMapper4.0 software (Applied Biosystems) and then compared with the ATCC, DSMZ or JCRB databases for reference matching.

To induce intracellular GSH depletion, cells were incubated in Cys_2_-free medium or with BSO (1000 μM) treatment or erastin (10 µM) treatment.

### GSH determination

GSH levels were determined using a colorimetric GSH assay kit according to the manufacturer’s instruction. Briefly, cellular pellets (10 µg) were mixed with 30 µl 5% metaphosphoric acid, and then frozen and thawed twice using liquid nitrogen and 37 °C water. The samples were centrifuged, and the supernatant was subjected to a GSH assay based on a kinetic enzymatic recycling method that detects the oxidation of GSH by 5,5′-dithiobis-2-nitrobenzoic acid (DTNB) and glutathione reductase to measure the GSH content in cells^74^. The absorbance was measured at 412 nm with a BioTek Synergy H1 hybrid Microplate Readers. GSH content were normalized to protein concentration and expressed as µM per mg protein.

### Cell viability detection with Annexin V/PI by flow cytometry

ARPE-19 cells were rinsed with PBS. The cell suspensions were washed twice with ice-cold PBS before staining using an Annexin V/PI staining kit (Alexa Fluor^®^ 488 annexin V/Dead Cell Apoptosis Kit). Briefly, 500 µl binding buffer was added to each tube and transferred to a 1.5 ml centrifuge tube (1–5×10^5^ cells). Then, 5 µl of Annexin V–FITC and 5 µl of propidium iodide (PI) were added, and the cells were gently vortexed. Cells were then incubated for 15 min at room temperature in the dark, and analyzed by flow cytometry (LSRFortessa^TM^; BD, Franklin Lakes, NJ, USA). Data were collected from at least 10,000 cells. The results were analyzed by FlowJo 7.6.2 software (Tree Star Inc., Ashland, OR, USA). Three independent experiments were conducted.

### Cell cycle analysis

For cell cycle analysis, cells were harvested and fixed in 80% ethanol overnight at −20 °C, washed with PBS, and then stained with PI and 100 μg/ml RNaseA. DNA content was measured by sorting the fluorescence activated cells by flow cytometry (LSRFortessa^TM^; BD, Franklin Lakes, NJ, USA). Data were collected from at least 10,000 cells. The results were analyzed by FlowJo 7.6.2 software (Tree Star Inc., Ashland, OR, USA). Three independent experiments were conducted.

### Measurement of ROS production

Cells were washed in prewarmed PBS and treated with 10 μM H_2_DCFDA for 30 min in dark. H_2_DCFDA is a nonfluorescent ester of the dye fluorescein that is cleaved by intracellular esterases and is entrapped within the cell as the oxidant sensitive DCF compound. ROS oxidize DCF to the fluorescent product fluorescein. The MFI was determined by flow cytometry (LSRFortessa^TM^; BD, Franklin Lakes, NJ, USA) in which DCF emission was recorded on channel FL1-H. Control cells were treated with H_2_O_2_ (1 mM) for 30 min as a positive control for increased ROS production. Data were collected from at least 10,000 cells. The results were analyzed by FlowJo 7.6.2 software (Tree Star Inc., Ashland, OR, USA). Three independent experiments were conducted.

### Detection of mitochondrial superoxide with MitoSOX

The production of superoxide in mitochondria was visualized with MitoSOX Red (Life Technologies). Confluent cells grown on six-well plate were subjected with indicated treatments for 24 h. Before termination of treatment, cells were incubated with 5 μM MitoSOX for 15 min at 37 °C, and washed in PBS. The MFI was determined by flow cytometry (LSRFortessa^TM^; BD, Franklin Lakes, NJ, USA). MitoSOX emission was recorded on channels FL2-H at 585 nm. Data were collected from at least 10,000 cells. The results were analyzed by FlowJo 7.6.2 software (Tree Star Inc., Ashland, OR, USA). Three independent experiments were conducted.

### Detection of lipid peroxidation

Peroxidation was examined by monitoring change in fluorescence emission of C11- BODIPY 581/591 from red to green^75^. Cells seeded in six-well plate were incubated with C11-BODIPY 581/591 at a final concentration of 10 µM for 30 min at 37 °C and washed three times with PBS. The MFI was determined by flow cytometry (LSRFortessa^TM^; BD, Franklin Lakes, NJ, USA) in which BODIPY emission was recorded on channels FL1-H at 530 nm and FL2-H at 585 nm. Data were collected from at least 10,000 cells. The results were analyzed by FlowJo 7.6.2 software (Tree Star Inc., Ashland, OR, USA). Three independent experiments were conducted.

### Estimation of mitochondrial mass

Mitochondrial mass was measured by MitoTracker dye as previously described^76^. Cells were loaded with MitoTracker Red dye (Excitation/Emission: 579/599 nm) at 100 nM final concentration (37 °C for 15 min) mounted in Live Cell Imaging Solution (Thermo Fisher), and viewed under a laser confocal microscope (LSM 510; Zeiss, Thornwood, NY, USA). Every experiment was repeated at least three times, and representative data are shown. ImageJ software (version 1.42q) was used for quantitative analysis of the MFI.

### Transmission electron microscopy

Cells were digested with 0.25% trypsin and 0.02% EDTA, washed with Hanks’ Balanced Salt Solution (HBSS). Pre-fixation was done with 2.5% glutaraldehyde phosphate (0.1 M, pH 7.4) overnight at 4 °C. Post-fixation proceeded in buffered osmium tetroxide, followed by dehydration before embedding in Epon812. Ultrathin sections (80 nm thick) were cut with an ultramicrotome (Leica, EMUC6, Germany), and then stained with uranyl acetate and lead citrate and finally examined by a Tecnai G^2^ Spirit TWIN transmission electron microscope (FEI, Hillsboro, OR, USA). For each condition, at least 100 cells from randomly chosen fields were observed.

### SAHF detection

Cells were fixed with 4% paraformaldehyde and washed with PBS. DAPI at 300 nM concentration in PBS was added for 5 min incubation. The cells were then washed three times with PBS, drained and mounted. DAPI-stained nuclei with blue fluorescence were viewed under laser confocal microscope (LSM 510; Zeiss, Thornwood, NY, USA).

### Analysis of oxidative stress-induced cellular senescence

Cell senescence assay was conducted with Senescence β-Galactosidase Staining Kit (Cell Signaling Technology) according to the manufacturer’s instruction. Hydrogen peroxide (100 μM, 90 min) was used as a positive control^10^. Early passage ARPE-19 cells were used as a negative control. Briefly, cells were seeded in six-well plate, growth media was removed from the cells before assay followed by a rinse with PBS. Cells were fixed with 1× Fixative Solution for 10 min at room temperature followed by a two-time rinse with PBS. Then 1 ml of the β-galactosidase staining solution was added to each well, and incubated at 37 °C overnight. Bright field mode was used to detect for the development of blue color under a Zeiss Axioscope microscope equipped with a Zeiss HRC microscope camera (×200 total magnification).

### RNA-seq and analysis

Total RNA isolation, cDNA library construction, and sequencing were performed at BGI (Beijing Genomics Institute, Shenzhen, Guangdong, China) using RNA-seq technology, as previously described^77,78^. High-quality reads were aligned to the human reference genome (UCSC_hg38). The expression levels for each of the genes were normalized to fragments per kilobase of exon model per million mapped reads (FPKM) using a software package called RSEM^79^. Three biological replicates were carried out in this study. DEGseq method was used to screen for differentially expressed genes ^80^.

### Western blotting

Cells were plated, grown to 70% confluency, and then subjected to indicated treatments. Cells were lysed at 4 °C in a radioimmunoprecipitation assay (RIPA) lysis buffer (Beyotime Biotechnology). Protein concentration was determined using a BCA method. Samples (30 µg protein) were resolved by 4–20% SDS-PAGE (Biofuraw™ Precast Gel, Tanon Science, Shanghai, China), transferred to polyvinylidene difluoride membranes, and incubated with one of the following antibodies: LC3 (88588s, Cell Signaling Technology, 1:1000), GPX4 (ab125066, Abcam, 1:1000), p16 (1003-1-AP, Proteintech, 1:1000). GAPDH (5174S, Cell Signaling Technology, 1:1000) was used as a loading control. Representative blots of at least two independent experiments are shown.

### Lentiviral transduction of mRFP-GFP-LC3 shRNA and autophagic flux determination

Lentiviruses encoding short hairpin RNAs (shRNA) to human mRFP-GFP-LC3-puro were constructed by Hanbio Biotechnology (Hanbio, Shanghai, China). ARPE-19 cells were seeded on a 24-well plate (1×10^5^ cells/well) for lentivirus transduction. Twenty-four hours later the culture medium was replaced with the lentivirus-containing medium (lentiviral titer 1×10^8^ TU/mL, MOI = 3) and incubated for 6 h. Upon virus transduction, the incubation medium was removed and fresh culture medium was added. Seventy-two hours after transduction, puromycin-containing medium (2 mg/mL) was used for selection. Fifty clones were pooled, expanded and used for experiments. For autophagic flux determination, cells were viewed under a laser confocal microscope (LSM 510; Zeiss, Thornwood, NY, USA), using a GFP and RFP filter to detect autophagosomes (yellow puncta) and autolysosomes (red puncta). ImageJ software (version 1.42q) was used for count yellow and red puncta.

### Statistical analysis

Statistical analysis was performed with SPSS 21.0 for Windows (IBM, Armonk, NY, USA). All data are expressed as the mean ± SD from at least three biological replicates, and comparisons between the two groups were performed with a nonparametric method (Mann−Whitney *U* test). A value of *p* < 0.05 was considered statistically significant.
